# Wound Healing: Molecular Mechanisms, Antimicrobial Peptides, and Emerging Technologies in Regenerative Medicine

**DOI:** 10.3390/ph18101525

**Published:** 2025-10-10

**Authors:** Ana Paula de Araújo Boleti, Ana Cristina Jacobowski, Breno Emanuel Farias Frihling, Maurício Vicente Cruz, Kristiane Fanti Del Pino Santos, Ludovico Migliolo, Lucas Rannier Melo de Andrade, Maria Ligia Rodrigues Macedo

**Affiliations:** 1 Protein Purification and Biological Functions Laboratory, Food Technology and Public Health Unit, Federal University of Mato Grosso do Sul (UFMS), Campo Grande 79070-900, Brazil; apboleti@gmail.com (A.P.d.A.B.); anacristinaj@gmail.com (A.C.J.); brenoemanuelfarias@gmail.com (B.E.F.F.); mauricio.cruz@ifg.edu.br (M.V.C.); kristianefdp@gmail.com (K.F.D.P.S.); rannier.andrade@outlook.com (L.R.M.d.A.); 2 S-Inova Biotech, Postgraduate Program in Biotechnology, Dom Bosco Catholic University, Campo Grande 79117-900, Brazil; rf4900@ucdb.br; 3 Federal Institute of Education, Science and Technology of Goiás, Goiania 74270-040, Brazil; 4 Department of Pharmaceutical Sciences, Food, and Nutrition, Federal University of Mato Grosso do Sul, P.O. Box 549, Campo Grande 79070-900, Brazil

**Keywords:** antimicrobial peptides, biofilm, gene therapy, tissue regeneration, nanotechnology, artificial intelligence

## Abstract

Wound healing is a dynamic process involving distinct phases that are regulated by cellular and molecular interactions. This review explores the fundamental mechanisms involved in wound healing, including the roles of cytokines and growth factors within the local microenvironment, with a particular focus on antimicrobial peptides (AMPs) as immune modulators and therapeutic agents in chronic wounds. Notably, AMPs such as LL-37 have been shown to reduce biofilm density by up to 60%, highlighting their dual role in both modulating host immune responses and combating persistent bacterial infections. It further examines emerging technologies that are transforming the field, extending beyond traditional biological mechanisms to innovations such as smart dressings, 3D bioprinting, AI-driven therapies, regenerative medicine, gene therapy, and organoid models. Additionally, the review addresses strategies to overcome bacterial biofilms and highlights promising approaches including biomaterials, nanomedicine, gene therapy, peptide-loaded nanoparticles, and the application of organoids as advanced platforms for studying and enhancing wound repair.

## 1. Introduction

All living organisms have developed mechanisms to repair cells and tissues after injury. Skin, the largest organ in the human body, is essential to this process. It acts not only as a mechanical barrier to prevent water loss but also as a key regulator of temperature, external stimuli detection, and the immune response [1]. Given its clinical importance, wound healing has been extensively investigated for over a century. Today, many of the cellular and molecular mechanisms that coordinate skin tissue repair are well-characterized, although ongoing research continues to reveal new insights [2].

Despite these advances, chronic wounds continue to pose a major and increasingly critical challenge to global public health. Unlike acute injuries, which generally heal spontaneously, chronic wounds persist and are often associated with underlying comorbidities. The elderly, diabetic patients, and individuals with reduced mobility are particularly vulnerable. These wounds can lead to serious infections, amputations, and even death [3].

The clinical relevance of chronic wounds remains substantially underrecognized. An alarming example is the diabetic foot ulcer, whose five-year mortality rate exceeds that of breast and prostate cancer. These data highlight a silent epidemic; one often neglected by both public policy and research agendas. A recent systematic review evaluated 28 randomized clinical trials on the use of herbal products for diabetic foot ulcer healing. Topical daily application of olive oil and oral intake of bitter melon leaf extract for 28 days significantly improved ulcer healing. Despite these promising results, the low methodological quality and heterogeneity of the studies underscore the need for more rigorous trials to draw definitive conclusions [4]. Consequently, there is an urgent need to increase awareness of the systemic impact of chronic wounds and develop more effective and integrated therapeutic strategies for their management [5].

Wound healing is a highly coordinated physiological process involving dynamic interactions between local and infiltrating cells, the extracellular matrix, and various cytokines. In an acute wound, this mechanism unfolds in sequential and overlapping steps (hemostasis, inflammation, proliferation, and remodeling) to control injury, eliminate pathogens, and restore tissue integrity [6]. In contrast, a chronic wound is characterized by a pathological disruption of this orderly progression. The healing process remains trapped in a state of chronic inflammation, failing to progress, unable to advance to the proliferative and remodeling phases. This arrest impairs restoration and leading to a vicious cycle of persistent damage, infection, and dysfunctional inflammation [2].

The challenge of tissue regeneration is particularly challenging in specialized tissues such as bone. Bone regeneration is a highly regulated, sequential process involving stages of inflammation, repair, and remodeling [7], where proper modulation of the initial acute inflammatory response is essential for success [8]. However, aging is associated with inflammaging, a state of chronic low-grade inflammation that progressively disrupts this regenerative cascade. This systemic inflammation increases susceptibility to diseases such as osteoporosis and diabetes and directly impairs fracture repair, complicating otherwise simple injuries into complex clinical conditions. Therefore, understanding and regulating the inflammatory response, particularly in the elderly, is a central therapeutic target for restoring regenerative capacity [9].

AMPs are a promising therapeutic strategy for chronic wounds due to their ability to control microbes, modulate immunity, and promote healing [10]. While some natural and synthetic AMPs have advanced to clinical trials for wound care [11], their transition to routine use is limited by their instability under environmental conditions. Key challenges within the wound microenvironment include pH alterations, proteolysis, hydrolysis, oxidation, and photolysis [10,12]. Overcoming these limitations is essential to unlocking the full clinical potential of AMPs.

The standard of care for tissue repair has evolved from passive wound coverings to active therapeutic platforms. Early dressings, such as cotton, served primarily as protection but often hindered healing by causing wound desiccation [13]. A paradigm shift occurred with the discovery that a moist environment significantly accelerates healing, leading to the development of modern dressings based on advanced biomaterials Natural polymers (collagen, chitosan, hyaluronic acid) and synthetic polymers are now engineered into hydrogels, films, and nanofibers. These sophisticated platforms maintain an optimal healing environment and can be engineered to release antimicrobial agents. At the same time, they mimic the native extracellular matrix (ECM) to support cell growth [14].

In bone regeneration, a major challenge is filling critical-sized defects that will not heal on their own. Biomaterials address this by serving as osteoconductive and osteo inductive scaffolds [15]. These scaffolds are composed of materials such as hydroxyapatite, calcium phosphates, and biodegradable polymers, and are engineered to provide mechanical support and guide the growth of new bone tissue. Just as with skin dressings, these scaffolds can be loaded with growth factors (BMPs) or stem cells to accelerate repair, demonstrating a similar tissue engineering approach used in soft tissue repair [16].

Innovation in both fields now focuses on merging these platforms with artificial intelligence (AI) to advance predictive regenerative medicine. For chronic wounds, AI algorithms analyze clinical or histological images to quantify healing, predict infection risks, and personalize the choice of dressing [17]. In bone regeneration, AI is used to design scaffolds with optimized architecture. It can also predict graft success from medical imaging and monitor bone consolidation. This synergy between reparative biomaterials and predictive, personalized approach is the most promising to overcoming the barriers of tissue regeneration [18].

This review aims to explore the cellular and molecular mechanisms involved in wound and fracture healing, with emphasis on the role of AMPs and their interaction with host immune responses. It focuses particularly on bacterial biofilm formation, a key factor in wound chronification. In parallel, it examines innovative and emerging therapies that are transforming the field of wound care. It goes beyond classical biological mechanisms to analyze disruptive innovations. These include exploring disruptive innovations, including smart dressings, 3D bioprinting scaffolds, AI-driven therapeutic platforms, and advanced strategies such as nanomedicine (peptide-loaded nanoparticles), gene therapy, and organoid models.

## 2. The Cellular and Molecular Landscape of Wound Healing

**Figure 1:**
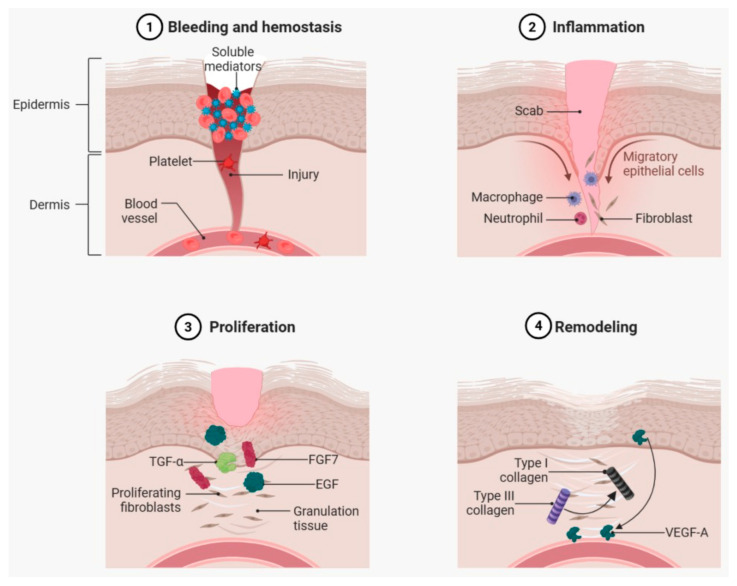
Phases of cutaneous wound healing. ① Clot formation: Initiates the healing process through platelet activation and the release of soluble mediators such as TGF-β, PDGF, SDF-1 (CXCL12), and VEGF. ② Inflammation: Tissue injury releases PAMPs and DAMPs—including HMGB1, hyaluronic acid, and ATP—that activate pattern recognition receptors (PRRs), such as Toll-like receptors (TLRs), RIG-I-like receptors, and cytoplasmic DNA sensors. ③ Proliferation: Growth factors including FGF7, EGF, and TGF-α, released by fibroblasts, platelets, and infiltrating macrophages, stimulate cell proliferation and the formation of new tissue. ④ Remodeling and angiogenesis: VEGF-A, released by epithelial cells at the wound edge and macrophages, promotes new blood vessel formation. Created in BioRender (Version: BioRender Premium Subscription), Academic Individual License granted to UCD/E. B. Souto OBRSS Research Support Scheme (AIUCD241022-cd0d). Souto, E. (2025) https://BioRender.com/tkp9loc (accessed on 9 August 2025).

Wound healing is a highly coordinated process, divided into sequential stages in which different cell types play crucial roles. Acute wound healing is a tightly regulated and efficient process, supported by compensatory mechanisms that prevent failure even in the face of minor disturbances [19]. The classification of skin wounds into acute and chronic skin is based on their pathogenesis and consequences. Acute wounds undergo a series of molecular events that eventually restore structural integrity. However, when more complex dysfunctions occur, the repair process can be compromised, resulting in abnormal healing or complete failure. In contrast, chronic wounds fail to resolve and are marked by pathological processes such as persistent inflammation, infection, and tissue necrosis. The phases include the formation of the initial clot, the inflammatory response, tissue regeneration (encompassing re-epithelialization and granulation tissue formation), and, finally, remodeling and resolution of the tissue (Figure 1) [19].

### 2.1. Clot Formation and Reepithelialization

The formation of a clot represents the first key phase of wound healing, providing a barrier to fluid loss and pathogen entry. When the vasculature is disrupted, blood and platelets extravasate, initiating platelet activation and the coagulation cascade. This process culminates in the conversion of fibrinogen to fibrin by thrombin, forming a fibrin mesh that seals the defect [20], a process primarily driven by the intrinsic coagulation pathway.

This pathway is activated when factor XII interacts with negatively charged surfaces, such as collagen, initiating a cascade of events that amplify clot formation. Deficiencies in this process can lead to continuous bleeding, impairing repair. In addition to forming the clot, platelets rapidly release soluble mediators such as TGFβ, PDGF, SDF1 (CXCL12), VEGF, and endostatin, which regulate the healing process [21]. Additionally, the coagulation cascade is closely linked to the innate inflammatory immune response, with both activating each other at the wound site. This interaction enhances the efficiency of the healing process during the early stages [22], setting the stage for subsequent re-epithelialization, a crucial process for cutaneous repair that involves cell proliferation and migration. Studies have revealed proliferative zones behind the migratory edge of the epidermis, which emerge hours after wounding and persist for several days. These zones expand as the wound size increases, with proliferative cells spreading into the central area after the edges of the wound fuse. Cell division is initially inhibited by cell–cell contact in the intact epidermal layer, but this inhibition is released following injury, facilitated by the mechanosensitive receptor PIEZO1, which regulates mitosis through calcium-dependent and ERK1/2 signaling [23]. Additionally, growth factors such as FGF7, EGF, and TGFα play crucial roles in stimulating cell proliferation, being released by fibroblasts, platelets, and infiltrating macrophages [24,25].

The process of re-epithelialization extends beyond mere cell division, as epidermal cells at the wound edge activate genes associated with adhesion, migration, and extracellular matrix (ECM) remodeling. During migration, keratinocytes alter the expression of integrins, deactivating hemidesmosome integrins such as α6β4 and activating others to facilitate movement through the provisional wound matrix [26]. Additionally, the cells produce proteases, such as matrix metalloproteinase 1 (MMP1), which cleave ECM components to allow dynamic adhesion and advancement. This coordinated process, involving proliferation, adhesion, and matrix remodeling, is essential for effective wound closure [27].

### 2.2. Damage Signaling in Wounds

Upon skin injury, a complex cascade of damage signals is triggered, including pathogen-associated molecular patterns (PAMPs) and damage-associated molecular patterns (DAMPs). Cellular lysis at the wound margin releases molecules such as HMGB1, hyaluronic acid, heat shock proteins, ATP, nucleosomes, and other components, which are recognized by pattern recognition receptors (PRRs), including Toll-like receptors (TLRs), RIG-I-like receptors, and cytoplasmic DNA sensors. These receptors activate immune cells and stimulate the production of pro-inflammatory cytokines, primarily via NF-κB signaling [28].

Additionally, wounds trigger rapid transcription-independent responses, such as an immediate Ca^2+^ wave and the release of reactive oxygen species (ROS) [29]. The increase in cytosolic Ca^2+^, initiated by changes in plasma membrane permeability, propagates through the tissue via gap junctions or by activating channels such as TRP. This Ca^2+^ influx activates NADPH oxidases and phospholipases, promoting the generation of ROS, such as H_2_O_2_, which facilitates the recruitment of inflammatory cells to the wound site [30]. Local signals, such as lipid peroxidation, can also act as long-range messengers, aiding in the detection of injury and guiding innate immune cells for tissue repair. This integrated interaction of signals and receptors ensures a coordinated response to damage [31].

### 2.3. Role of Neutrophils and Macrophages in the Wound Healing Process

The process of wound healing involves the coordinated activity of diverse immune cells, such as neutrophils and macrophage. These cells play a critical role in the initial inflammatory response, angiogenesis and tissue remodeling. These processes are essential for protection against infection, tissue repair and restoration of skin integrity [18].

At the onset of injury, neutrophils are the first immune cells to reach the wound site. This process begins with the extravasation of cells from the bloodstream into the injured tissue. Initially, they loosely adhere to the inflamed endothelial walls through selectin-mediated interactions, which slow their movement and allow them to spread and traverse the endothelium and pericytes (diapedesis) [32] setting the stage for directed migration towards the wound, guided by damage signals, pathogen-associated molecular patterns (PAMPs), damage-associated molecular patterns (DAMPs), and chemokines such as CXCL8 and LTB4 [33]. These signals activate specific receptors, such as CXCRs, and promote GTPase-regulated migration [34]. During this process, neutrophils release autocrine and paracrine signals that amplify recruitment and generate swarming behavior, which is mediated by LTB4 and integrins. In some species, this behavior is also associated with the formation of neutrophil extracellular traps (NETs) [35].

At the injury site, the primary function of neutrophils is to combat infections. They eliminate microorganisms through the targeted release of reactive oxygen species (ROS) and the formation of NETs, which consist of extracellular DNA associated with antimicrobial proteins. This process depends on molecules such as neutrophil elastase, peptidylarginine deiminase 4 (PAD4), and gasdermin D [36], and it also helps shape the inflammatory environment that subsequently recruits and activates macrophages. Macrophages play multiple roles in wound healing, adjusting their function according to the needs of the tissue at different stages of the process. Initially, they promote inflammation by releasing pro-inflammatory cytokines and attracting more immune cells to the wound site [37]. As healing progresses, they modulate their activity to promote angiogenesis, stimulating the growth of new blood vessels necessary for supplying oxygen and nutrients to the repairing tissue. They also participate in the deposition of the extracellular matrix and the regulation of scar formation, playing a crucial role in the transition to the remodeling phase and the resolution of the inflammatory process. Together, neutrophils and macrophages coordinate the inflammatory and repair responses, ensuring a balance between defense against infection and tissue regeneration [37].

Derived from monocytes originating from different sources, they are activated by the release of DAMPs shortly after injury and recruit other macrophages through chemoattractant such as MCP1 and lipid mediators like LTB4. At the injury site, they phagocytize cellular debris, regulate the extracellular matrix (ECM) and interact with fibroblasts to promote tissue remodeling [38,39].

During healing, macrophages transition from a pro-inflammatory (M1) phenotype, predominant in the early stages, to an anti-inflammatory (M2) phenotype in later phases. This transition, driven by signals such as IL-4 and IL-13, is crucial for the resolution of inflammation and tissue regeneration [40]. The efferocytosis of apoptotic neutrophils plays a central role in this process, stimulating the production of lipoxins, which terminate inflammation and promote the recruitment of monocytes and tissue repair [37].

Additionally, macrophages influence various cell lineages, such as fibroblasts, endothelial cells, and adipocytes, adapting their functions according to the stage of repair. Studies show that the depletion of macrophages at different points in the process can impair angiogenesis, re-epithelialization, and wound closure, highlighting their importance in the dynamic and coordinated control of healing [39].

### 2.4. Angiogenesis in Wound Healing

Skin injuries often damage the local vasculature, which must be restored and expanded to support the increased metabolic and oxygen demands during tissue repair, thereby coordinating with subsequent phases of wound healing. This process, known as angiogenesis, forms a dense capillary network, referred to as “granulation tissue” [41]. The process begins within hours after the injury, with the sprouting of vascular tips that result in a dense capillary bed. VEGFA, released by epithelial cells at the wound edge and macrophages, is the main pro-angiogenic factor [2].

The absence of macrophages in the early stages impairs vascular formation, while their persistence in a pro-inflammatory state prevents the resolution and pruning of vessels. In addition to VEGFA, factors such as FGF2 and PEDF influence the angiogenic process [42]. Lymphatic vessels are also essential in repair, playing roles in fluid homeostasis and immune surveillance. After injury, lymphangiogenesis occurs through the expansion of pre-existing lymphatic vessels, driven in part by signals from macrophages. Key mediators include VEGFR3 and its ligands VEGFC and VEGFD [43].

### 2.5. Remodeling

Wound healing undergoes a prolonged phase of tissue remodeling or maturation, which can last for several months or even years. This period is crucial in determining the likelihood of scar formation and the risk of wound recurrence [18], during which granulation tissue is gradually replaced by scar tissue and the neo-vasculature regresses. Initially, granulation tissue is rich in type III collagen, which is gradually replaced by type I collagen [44]. During this process, myofibroblasts produce MMPs and their inhibitors (TIMPs), which are essential for the selective degradation of extracellular matrix (ECM) components. However, an imbalance between MMPs and TIMPs can lead to alterations in ECM and promote the development of chronic wounds [45].

During this stage, macrophages are crucial for degrading excess ECM and removing cellular debris via phagocytosis. Meanwhile, the blood vessels formed during angiogenesis exhibit high permeability and weak intercellular junctions, facilitating the infiltration of immune cells [45]. The stabilization of these vessels depends on the pruning process, which eliminates unnecessary neo vessels through apoptosis of endothelial cells (ECs). Re-epithelialization also influences this stage [46]. In addition, endothelial cells have self-regulatory mechanisms, such as the Sprouty and Vasohibin proteins, which modulate their response to VEGF, while the expression of CXCR3 in the later stages inhibits endothelial tube formation [22]. Understanding and controlling these processes paves the way for more effective therapies, promoting faster healing with fewer scars.

### 2.6. Failures in Tissue Repair

**Figure 2:**
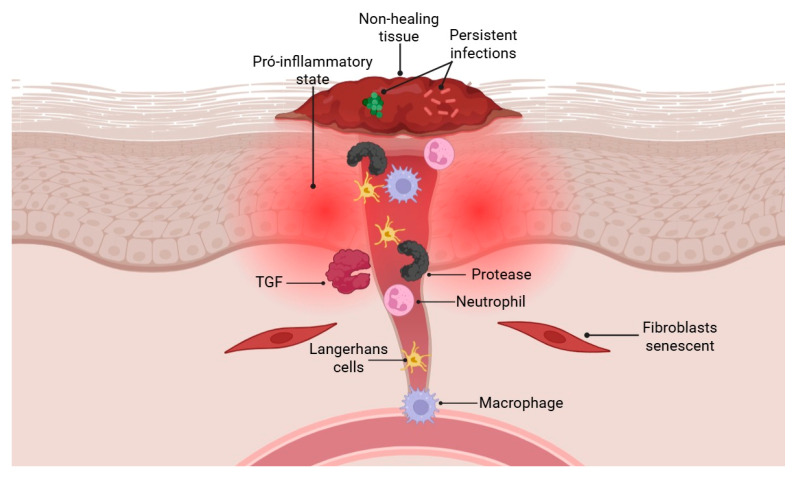
Key pathological mechanisms in non-healing tissue. Cellular senescence contributes to chronic inflammation through the secretion of pro-inflammatory cytokines and proteases. Persistent immune cell infiltration (e.g., neutrophils, pro-inflammatory macrophages) and elevated protease activity degrade extracellular matrix (ECM) components, growth factors (e.g., VEGF, TGF-β), and cytokines (e.g., TNF-α). Senescent fibroblasts exhibit impaired ECM deposition and reduced responsiveness to regenerative signals, further hindering tissue repair. Created in BioRender, Academic Individual License granted to UCD/E. B. Souto OBRSS Research Support Scheme (AIUCD241022-cd0d). Souto, E. (2025) https://BioRender.com/7ftaa82 (accessed on 1 August 2025).

Acute wound healing is a tightly regulated and efficient process, supported by compensatory mechanisms that prevent failure even in the face of minor disturbances. However, when more complex dysfunctions occur, the repair process can be compromised, resulting in abnormal healing or complete failure (Figure 2) [18]. Wounds that remain open for more than 12 weeks are classified as chronic and are common among the elderly and individuals with diabetes [47]. These wounds pose a significant clinical challenge due to their impact on quality of life and the high associated healthcare costs. The resistance of many of these wounds to available treatments underscores the need for a deeper understanding of the cellular mechanisms that regulate tissue repair to develop more effective therapeutic strategies [48].

Elderly and diabetic individuals are more susceptible to skin injuries due to cutaneous atrophy, barrier dysfunction, and dermal matrix degradation, which collectively weakens tissue integrity [49,50]. Following injury, several factors contribute to healing failure. Among these, cellular senescence plays a central role: mitotic cells cease to proliferate and begin secreting pro-inflammatory cytokines and tissue-degrading proteases, exacerbating the inflammatory environment. This is further intensified by high oxidative stress and persistent inflammation, which are hallmark features of chronic wounds [51].

Excessive inflammation is another key factor in wound chronicity. Non-healing wounds are heavily infiltrated by immune cells such as neutrophils, Langerhans cells, and pro-inflammatory macrophages, along with high levels of proteases—all of which are associated with clinical severity. Neutrophils are hyperactive, releasing cytotoxic extracellular traps and showing resistance to apoptosis. Diabetic macrophages are also dysfunctional, exhibiting impaired phagocytosis, ineffective efferocytosis, and difficulty transitioning to an anti-inflammatory phenotype [52].

Even before ulceration, diabetic skin shows a pro-inflammatory state, with increased numbers of mast cells and activated macrophages. In contrast, T cell receptor diversity and CD4^+^ T cell counts are reduced, weakening the adaptive immune response. These immune imbalances impair inflammation resolution and promote chronic infection, creating a vicious cycle of tissue damage, inflammation, and ineffective repair [53].

Wound healing impairment in chronic wounds extends beyond inflammation to include defects in re epithelialization and dermal remodeling. Diabetic foot ulcers often display hyperkeratotic epidermal edges, where keratinocytes exhibit molecular changes such as nuclear accumulation of β-catenin and overexpression of c-myc, which delay cell migration and wound closure. The epidermis in these regions also shows abnormal expression of cell cycle and differentiation markers, impaired growth factor signaling, and absence of hair follicles—features that reflect an aberrant activation phenotype detrimental to healing [2].

In the dermis, remodeling is hindered by high protease levels that degrade extracellular matrix (ECM) components, growth factors (e.g., VEGF, TGF-β), and cytokines (e.g., TNF-α). Fibroblasts in chronic wounds are often senescent, with reduced capacity to deposit and organize ECM, and show diminished responsiveness to stimulatory signals such as TGF-β. Iron availability emerges as a relevant factor: iron deficiency reduces ECM deposition, while intracellular iron loading promotes matrix remodeling and the activation of reparative macrophages [54].

In diabetes, sustained hyperglycemia further impairs wound healing by disrupting leukocyte function, inducing cellular senescence, and promoting the formation of advanced glycation end-products (AGEs), which alter dermal architecture and trigger inflammation via the RAGE receptor [55]. These effects hinder neovascularization and cause long-term damage to the microvasculature, leading to local hypoxia, arterial vasculopathy, and neuropathy—all of which increase the risk of chronic wounds [56]. Chronic wounds are multifactorial, involving hormonal imbalances and altered growth factors, such as decreased platelet-derived growth factor. In addition, persistent infections by pathogens such as *Staphylococcus aureus* and *Pseudomonas aeruginosa*, often organized in antibiotic-resistant biofilms, exacerbate healing impairment in diabetic wounds [57].

### 2.7. Fracture Healing

Fracture healing is a highly coordinated process involving molecular, cellular, and tissue events, whose proper progression depends on a stable biomechanical environment. Minimally displaced fractures, such as stress fractures, can heal through intramembranous ossification (primary bone formation). However, most fractures exhibit some degree of movement at the injury site, which favors endochondral ossification (secondary bone formation), the more common mechanism of consolidation [58].

This process is traditionally divided into four overlapping stages: inflammation, soft callus (cartilaginous callus) formation, hard callus (bony callus) formation, and remodeling. Each phase represents a set of continuous and interdependent events that can coexist in different regions of the fracture callus. Failure to progress through any of these stages may result in delayed healing or nonunion [59]. A recent study investigated the dynamics of the immune response and cytokine release on the third day post-fracture, corresponding to the initial inflammatory phase. Structural and functional outcomes were also evaluated during hard callus formation (days 14 and 21) and at the end of remodeling (day 35), providing an integrated view of the temporal progression of bone repair [60].

Fracture healing depends on a well-regulated initial inflammatory response, which begins with the formation of a fibrin-rich hematoma that acts as a scaffold for cellular recruitment and cytokine release. This inflammatory response occurs within the first hours and days and is essential for successful bone consolidation. Neutrophils are the first leukocytes to infiltrate the site, where they phagocytose pathogens and debris. Subsequently, pro-inflammatory monocytes and macrophages (M1) arrive between 24 and 48 h and secrete cytokines such as IL-1, IL-6, TNF-α, and CCL2, which are crucial for advancing the repair process [61].

Moreover, resident bone macrophages, known as osteomacs, play a direct role in osteogenesis. The interaction between neutrophils and macrophages is critical; neutrophil depletion, for example, leads to increased macrophage numbers and impairs the quality of the bony callus. Alterations during this early inflammatory phase are associated with reduced callus volume and mechanical strength, as observed in experimental models on day 21 post-fracture. Thus, the balance between initiation and resolution of inflammation is essential for effective bone healing [62].

Macrophages are key players in fracture consolidation, functioning both in clearing the injury site and modulating inflammation and cartilage and bone formation. The shift from the M1 (pro-inflammatory) phenotype to the M2 (regenerative) phenotype is vital for successful healing. Macrophage depletion in experimental models impairs granulation tissue and cartilaginous callus formation, resulting in delayed ossification and mechanically and structurally deficient calluses. Conversely, immunomodulatory cytokines such as CSF-1, IL-4, and IL-13 increase macrophage numbers (especially M2) and improve healing [63].

In addition to macrophages, T cells also actively participate in repair. For example, γδ T cells produce IL-17A, which promotes bone formation. Meanwhile, αβ T cells are activated by antigens presented via MHC, whereas γδ T cells respond to inflammatory cytokines such as IL-1β, IL-18, and IL-23, amplifying the immune response. Therefore, the balance and proper function of these cells are critical determinants for effective fracture consolidation [64,65].

## 3. Antimicrobial Peptides in Wound Healing

However, the presence of microbial infection represents a significant challenge, frequently leading to wound chronicity and severe complications [66]. In this context, AMPs emerge as promising multifunctional actors. Originally recognized as key components of innate immunity, capable of eliminating a broad spectrum of pathogens, AMPs demonstrate additional properties crucial for tissue repair, including modulation of the inflammatory response, promotion of angiogenesis, and stimulation of epithelial and stromal cell migration and proliferation [67,68].

### 3.1. Modulation of Innate Immunity and Direct Antimicrobial Action

AMPs are central effectors at the interface between defense against infection and the initiation of the tissue repair process. Their direct antimicrobial action is often mediated by interaction with microbial membranes, which are rich in anionic components such as lipopolysaccharides (LPS) in Gram-negative bacteria or lipoteichoic acid in Gram-positive bacteria [67]. The cationic and amphipathic nature of AMPs, such as the human cathelicidin LL-37, allows their insertion into and destabilization of these membranes, leading to cell lysis. Furthermore, many AMPs exhibit intracellular mechanisms, inhibiting the synthesis of essential macromolecules or microbial metabolic processes [69].

Parallel to their microbicidal activity, AMPs play a sophisticated role in modulating the innate immune response, essential for controlling inflammation and initiating the proliferative phase of healing. LL-37, for instance, can neutralize bacterial LPS, preventing excessive activation of receptor Toll-like 4 (TLR4) and the subsequent massive release of pro-inflammatory cytokines [70]. Recent studies indicate that LL-37 can also promote the activation of the NLRP3 inflammasome in macrophages under certain conditions, a process important for host defense but requiring fine regulation to prevent tissue damage [71]. Other AMPs, such as hepcidin, influence iron availability, an essential nutrient for microbes, and simultaneously modulate macrophage polarization, favoring M2 phenotypes associated with inflammation resolution and tissue repair [71,72].

### 3.2. Regulation of Cell Proliferation and Differentiation

AMPs transcend their defensive function, acting as important signaling molecules that directly influence the behavior of host cells involved in tissue regeneration. They can promote the migration, proliferation, and differentiation of keratinocytes, fibroblasts, and endothelial cells, accelerating wound closure and the restoration of the epithelial barrier. Human β-defensin 2 (hBD-2), for example, has been shown to stimulate keratinocyte migration through the activation of the epidermal growth factor receptor (EGFR), a crucial mechanism for re-epithelialization [73]. This activation can occur both directly and indirectly, through the modulation of other cellular signaling pathways.

Other endogenous AMPs exhibit equally relevant effects on the cellular matrix and angiogenesis. Histatins, found in saliva, accelerate healing in vitro and in vivo by promoting the migration of fibroblasts and epithelial cells, possibly through interaction with integrin receptors [74]. LL-37 also demonstrates pro-angiogenic activity, stimulating the formation of new blood vessels, a vital process for supplying nutrients and oxygen to the repairing tissue. Synthetic peptides derived from natural AMPs, such as IDR-1018, have been developed to optimize these immunomodulatory and pro-regenerative properties, demonstrating the capacity to induce chemotaxis of immune cells and promote healing in preclinical models [68].

### 3.3. Therapeutic Applications in Chronic Wounds

The unique ability of AMPs to combat infections, including those caused by antibiotic-resistant bacteria and biofilms (a major challenge detailed in Section 4), while simultaneously modulating inflammation and promoting tissue regeneration, positions them as promising therapeutic candidates for chronic wounds, where conventional treatments often fail [67,68]. Wounds such as diabetic foot ulcers, pressure ulcers, and venous ulcers are frequently colonized by microbial biofilms and characterized by chronic inflammation and impaired healing, representing a significant clinical challenge AMPs offer a multifaceted approach to break this vicious cycle [66].

Several AMPs and their synthetic analogs are being investigated in preclinical and clinical studies for wound treatment. The peptide PXL150, for instance, derived from amphibian esculentin-1a, has shown potent activity against bacterial biofilms relevant in diabetic foot ulcers [75,76]. Formulation strategies, such as incorporating AMPs into hydrogels, scaffolds, or nanoparticles, are crucial for improving stability, controlling release, and optimizing therapeutic efficacy in the complex wound environment. LL-37 analogs, such as DPK-060, have also shown encouraging results in promoting healing and reducing bacterial load in animal models and initial clinical studies for different types of wounds [77].

### 3.4. Limitations, Challenges and Strategies

Despite their potential as therapeutic agents in wound healing, the clinical application of antimicrobial peptides (AMPs) is limited by their instability in the wound microenvironment [78]. The presence of proteases and other degradative enzymes in wound exudate, coupled with the activity of the lymphatic system, can result in the rapid degradation of AMPs [79]. This compromises their efficacy and reduces their half-life at the site of action [80]. This requires the development of formulation strategies that protect the peptides from degradation and ensure their therapeutic persistence. Additionally, the toxicity of some AMPs at high concentrations and their high molecular mass can hinder diffusion and penetration into tissues, thereby limiting bioavailability and action at the site of infection [81].

To overcome these challenges, delivery strategies and structural modifications have been investigated. Incorporating AMPs into hydrogels, scaffolds and nanoparticles has emerged as an effective way of controlling stability and release, as well as optimizing therapeutic efficacy in the wound environment [82]. These formulations can protect peptides from enzymatic degradation, prolong their residence time at the wound site and facilitate their penetration into biofilms [83]. Furthermore, the chemical modification of AMPs, such as cyclisation or the incorporation of non-natural amino acids, can enhance their resistance to proteolysis and improve their pharmacokinetic properties [84,85].

## 4. Biofilms in Chronic Wounds

Chronic wounds represent a global clinical challenge of remarkable complexity, with their pathogenesis being intrinsically linked to persistent bacterial colonization and a chronic inflammatory state that impedes tissue resolution [56]. Among the central mechanisms underlying chronicity, the formation of bacterial biofilms stands out—structurally organized microbial consortia encapsulated within a polymeric extracellular matrix (EPS) that functions as a physicochemical barrier. These microbial communities exhibit multifactorial antimicrobial resistance while actively modulating innate immune responses, thereby creating an ecological niche that facilitates pathogen persistence even in hostile environments [86].

Biofilms exert active modulation of the innate immune response, inducing a dysfunctional immune activation state characterized by overexpression of pro-inflammatory cytokines (IL-1β, TNF-α) and suppression of reparative mediators (IL-10, TGF-β). This immune dysregulation maintains a chronic inflammatory microenvironment that inhibits the transition to the proliferative phase of wound healing through persistent M1 macrophage activation and impaired M2 polarization [87]. This pathophysiology necessitates combined therapeutic strategies that integrative address: (1) disruption of the biofilm extracellular matrix, (2) restoration of local immune homeostasis, and (3) stimulation of endogenous tissue repair mechanisms.

Prospective clinical studies employing advanced confocal microscopy and genomic sequencing techniques demonstrate that bacterial biofilms colonize over 60% of chronic wounds, with particularly high prevalence in diabetic foot ulcers, venous leg ulcers, and pressure injuries [88]. Biofilm-mediated clinical recalcitrance results in treatment failure rates reaching 78% with conventional protocols, contributing to devastating socioeconomic impact: global estimates indicate 178 million affected patients (2.1% of the world population), with annual direct costs of $27.5 billion [89]. This clinical reality has driven translational research focused on innovative anti-biofilm strategies, including photodynamic therapy, functionalized nanoparticles, and quorum-sensing inhibitors, which aim to overcome the extracellular matrix barrier and restore tissue healing [90].

### 4.1. Mechanisms of Formation and Resistance of Bacterial Biofilms in Chronic Wounds

The formation of bacterial biofilms in chronic wounds constitutes a dynamic and highly regulated process, conferring microbial communities with remarkable resistance to both antimicrobial agents and host immune responses [91].

The initial adhesion of pathogenic bacteria to biological or necrotic surfaces in chronic wounds constitutes a precisely regulated biophysical process that initiates a cascade of events leading to biofilm-associated infection and impaired wound healing [92]. This adhesion phenomenon is governed by an intricate interaction between bacterial surface determinants (e.g., adhesins, fimbriae, and cell wall teichoic acids) and physicochemical properties of the host tissue (surface free energy, nanotopography, and extracellular matrix composition) [93].

Following adhesion, bacteria initiate the secretion of EPS, whose composition includes polysaccharides (such as alginate, Pel and Psl in *Pseudomonas aeruginosa*), extracellular DNA (eDNA), proteins and lipids, which organize into a structurally consolidated three-dimensional matrix [94]. The biofilm maturation process is characterized by the formation of structurally and metabolically heterogeneous microcolonies, where the generation of nutritional and oxygen gradients establishes distinct microenvironments, promoting bacterial resistance to adverse environmental conditions [95].

The resistance of bacterial biofilms is intrinsically associated with the integrated action of efflux pumps and stress response pathways [96]. In *P. aeruginosa*, the MexAB-OprM efflux system plays a critical role in intrinsic resistance, actively expelling antibiotics such as β-lactams and fluoroquinolones from the cytoplasm, thereby reducing their intracellular concentration below the effective threshold. This mechanism, dependent on ATP hydrolysis, constitutes one of the main contributors to antimicrobial resistance in biofilms. Simultaneously, the stress response (stringent response), mediated by the alarmone (p)ppGpp, induces global metabolic reprogramming, including suppression of protein biosynthesis and generation of metabolically quiescent persistent cells (persisters) a phenotype crucial for antibiotic tolerance [97,98].

Furthermore, this pathway positively regulates the expression of efflux pumps, establishing a coordinated defense system. The synergy between these mechanisms—where efflux pumps confer constitutive resistance while the stress response enables dynamic adaptation—explains the remarkable resilience of biofilms to antimicrobial agents. This complex interaction represents a critical therapeutic challenge in the management of biofilm-associated infections [99].

Another critical aspect is the intercellular communication system mediated by quorum sensing (QS), which coordinates virulence factor production and biofilm dispersal. Gram-negative bacteria use acyl-homoserine lactones (AHLs) as signaling molecules, while Gram-positive species employ autoinducing peptides, both enabling population-wide gene expression coordination [99]. The release of planktonic cells from mature biofilms—facilitated by extracellular hydrolases such as eDNA-specific DNase I—promotes colonization of new niches, thereby facilitating infection dissemination [100].

In summary, biofilm formation and antimicrobial resistance represent multifactorial processes involving physical barriers (EPS matrix), metabolic adaptations (formation of persistent cells), and genetic regulation mechanisms (QS-controlled gene networks). A detailed understanding of these complex interactions is essential for developing targeted therapeutic strategies capable of overcoming the remarkable resilience of these structured microbial communities.

### 4.2. Impact of Biofilms on Chronic Inflammation and Delayed Healing

Microbial biofilms play a fundamental role in perpetuating chronic inflammation by directly disrupting immune regulation and tissue regeneration processes [19]. The persistent presence of biofilms triggers sustained activation of intracellular signaling complexes, particularly the NLRP3 inflammasome, resulting in excessive release of interleukin-1β (IL-1β)—a central pro-inflammatory cytokine that maintains chronic inflammatory states [101]. This NLRP3-predominant activation pattern is particularly relevant in chronic wounds, where biofilm persistence correlates with elevated levels of these cytokines and impaired tissue repair.

Hyperactivated neutrophils in the inflammatory microenvironment initiate intense formation of neutrophil extracellular traps (NETs). These structures, composed of decondensed DNA fibers associated with granular proteins (such as histones, elastase, and myeloperoxidase), represent a complex immune response with paradoxical effects [102]: (a) Protective Effect: efficient capture and neutralization of extracellular pathogens; creation of a physical barrier against microbial dissemination. (b) Deleterious Effect: induction of direct tissue damage through histone-mediated cytotoxicity, activation of matrix metalloproteinases (MMP-9), endothelial injury and microvascular barrier disruption, depletion of functional neutrophils, and generation of nuclear autoantigens.

Recent studies demonstrate that in *Staphylococcus aureus* bacteremia, NET formation occurs predominantly via activation of the TLR2/PAD4 axis [103] and NADPH oxidase-dependent production of reactive oxygen species (ROS). Paradoxically, while containing infection locally, NETs may increase vascular permeability, facilitate bacterial invasion into deeper tissues, and promote microvascular thrombosis through interactions with platelets and fibrinogen [104].

Beyond inducing immune dysregulation, bacterial biofilms significantly compromise tissue healing processes through multiple molecular mechanisms. Studies show a marked reduction in transforming growth factor-β (TGF-β) expression, a cytokine essential for fibroblast activation and proliferation—cells crucial for proper extracellular matrix synthesis and wound contraction. This decreased TGF-β signaling is directly associated with deficient granulation tissue formation [105] and reduced collagen deposition [106]. Povidone-iodine (PVI) interventions show promise, with evidence indicating that topical application can restore TGF-β levels in acute cutaneous wounds, promoting more efficient healing through stimulation of fibroblast proliferation and angiogenesis [107].

MMP-9, a zinc-dependent metalloproteinase, shows elevated activity in biofilm-colonized wounds, impairing healing through multiple mechanisms [108]. Its proteolytic action degrades key extracellular matrix components (collagen IV, fibronectin, and elastin), disrupting tissue architecture and hindering keratinocyte migration. Furthermore, MMP-9 inactivates essential growth factors (EGF, TGF-β, and PDGF) and generates matrix fragments that perpetuate inflammation. This proteolytic imbalance sustains a vicious cycle that promotes wound chronicity, positioning MMP-9 as a potential therapeutic target for treating biofilm-colonized wounds [105].

### 4.3. Advanced Therapeutic Approaches to Combating Biofilms Associated with Inflammation and Chronic Skin Wounds

Treatment of chronic infections associated with bacterial biofilms remains one of the greatest challenges in contemporary clinical practice, exacerbated by the growing scenario of antimicrobial resistance and the limited efficacy of conventional therapies. The EPS matrix acts as a multifunctional barrier, significantly reducing the penetration of antimicrobial agents (up to 1000-fold in some cases) and providing protection against host defense mechanisms, resulting in persistent and recalcitrant infections [97]. Facing this scenario, new therapeutic strategies have been developed based on four fundamental pillars [33,109]: biofilm matrix disruption, potentiation of antimicrobial activity, immune response modulation through QS inhibitors and inflammation regulators, and stimulation of tissue regeneration via growth factors and cell therapy.

**Figure 3:**
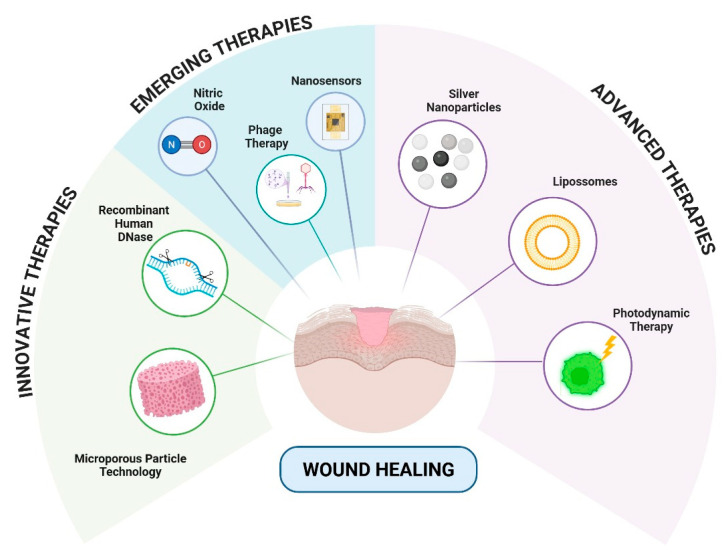
Schematic representation of the types of technologies developed for wound treatment. Created in BioRender, Academic Individual License granted to UCD/E. B. Souto OBRSS Research Support Scheme (AIUCD241022-cd0d). Souto, E. (2025) https://BioRender.com/hsuvhg7 (accessed on 9 August 2025).

The AMPs represent a particularly promising class of anti-biofilm agents due to their multifunctional mechanism of action. Unlike conventional antibiotics, many cationic AMPs can electrostatically interact with and disrupt the anionic components of the biofilm EPS (extracellular polymeric substance), facilitating deeper penetration. Furthermore, their ability to target bacterial membranes makes them effective against metabolically dormant persister cells within the biofilm, a significant challenge for many traditional agents. The development of synthetic AMP analogs and their incorporation into advanced delivery systems (hydrogels, nanoparticles) to enhance their stability and efficacy in the wound environment is a rapidly advancing field, as detailed in Section 3.3. Figure 3 represents some types of technology that are under development for more effective treatment of wounds.

#### 4.3.1. Innovative Therapies

In the context of targeted biological therapies, dornase alfa (recombinant human DNase, Pulmozyme^®^) has demonstrated clinical efficacy in degrading eDNA, a fundamental structural component of biofilm matrices. A case study showed that administration of dornase alfa (2.5 mg twice daily via nebulization) reduces biofilm density by up to 60% in chronic wounds colonized by *P. aeruginosa* [110].

In addition to promoting mechanical biofilm dispersion through hydrolysis of eDNA networks, this therapeutic approach significantly reduces (*p* < 0.01) the formation of NETs, structures that contribute to the perpetuation of local inflammatory responses and tissue damage. Confocal microscopy analyses revealed that treatment with dornase alfa decreases NET deposition by 45% in infected diabetic wounds, correlating with reduced levels of IL-1β and TNF-α in perilesional tissue [111].

#### 4.3.2. Advanced Therapies

Among the most well-established therapeutic strategies, silver nanoparticles (AgNPs) stand out as fundamental active components in next-generation antimicrobial dressings, accelerating granulation tissue formation in infected wounds [112,113]. The mechanism of action of AgNPs is based on the controlled release of Ag^+^ ions, which induce bacterial oxidative stress through the production of reactive oxygen species (ROS) and cause irreversible structural damage to the plasma membrane. Electron microscopy studies have demonstrated that AgNPs with an average diameter of 20 nm efficiently penetrate biofilm matrices, reaching intracellular bacterial concentrations significantly higher than those achieved with conventional ionic silver [114].

Notably, beyond their antimicrobial activity, AgNPs stimulate the proliferation of human fibroblasts in vitro. Multiple studies show that AgNPs are non-toxic to normal human fibroblasts and can enhance their migration and proliferation compared to untreated controls. For instance, AgNPs synthesized using plant extracts or incorporated into nanofiber structures significantly increased fibroblast proliferation and wound closure rates in scratch assays, with some studies reporting up to 96% wound closure in vitro [115,116,117,118].

Liposomal systems represent another frontier in biofilm infection treatment, overcoming the pharmacokinetic limitations of conventional antibiotics. Liposomal gentamicin formulations, with an average particle size of 150 nm and a positive surface charge, have demonstrated superior penetration and efficacy against microorganisms embedded in biofilms. This technology enables sustained tissue concentrations for up to 72 h after a single application, with Phase III clinical studies showing a 90% reduction in *P. aeruginosa* bacterial load in diabetic ulcers [119,120].

Antimicrobial photodynamic therapy (aPDT) has emerged as a promising modality, combining natural photosensitizers (such as >95% purity curcumin) with specific wavelength light sources (blue light). The photophysical mechanism involves ROS generation, particularly singlet oxygen (^1^O_2_), with a quantum yield of 0.25 under physiological conditions. In vivo assays using cutaneous infection models demonstrated that aPDT reduces bacterial viability while favorably modulating cytokine profiles, with a 60% decrease in IL-6 levels and a 2.5-fold increase in TGF-β1 expression [121]. This dual action—antimicrobial and immunomodulatory—positions aPDT as an integrative strategy in managing complex wounds [122].

#### 4.3.3. Emerging Therapies

Nitric oxide (NO) stands out as an endogenous signaling molecule with multifaceted therapeutic applications in biofilm infection treatment. As an antimicrobial agent, NO exerts biocidal action through the S-nitrosylation of essential proteins and induction of bacterial DNA damage, demonstrating efficacy against resistant pathogens such as *Staphylococcus aureus* and *P. aeruginosa* in in vitro models [123]. Innovative controlled-release systems, including silica nanoparticles functionalized with S-nitrosothiol (RSNO) groups and photoactivatable hydrogels containing NO precursors (nitrite or nitroprusside), have demonstrated the ability to reduce bacterial viability in mature biofilms, disperse the extracellular matrix through polysaccharide and eDNA degradation, and modulate the inflammatory response [124]. At physiological concentrations (picomolar to nanomolar), NO promotes angiogenesis via VEGF/VEGFR2 pathway activation, increasing vascular density by 30–50% in chronic wound models [93]. Preclinical studies using NO-releasing hydrogels showed significant acceleration (*p* < 0.01) of infected wound closure, with simultaneous bacterial load reduction and improved granulation tissue organization [125].

Among personalized therapeutic approaches, phage therapy, the use of strictly selected lytic bacteriophages emerges as a promising alternative in the face of increasing antimicrobial resistance [126]. Recent studies demonstrate that polyclonal phages, when characterized through whole-genome sequencing and host-range testing, exhibit unique capabilities to efficiently penetrate biofilm matrices via the expression of specific polymerases (such as endolysins and EPS-depolymerases) [121], selectively replicate within target bacterial populations, and induce programmed bacterial lysis with the release of new virions capable of infecting adjacent cells [127]. Furthermore, enzybiotics—phage-derived endolysins—have proven effective in disrupting Gram-positive bacterial cell walls, showing promise for topical use [128].

Among innovative therapeutic strategies, microporous particle technology (MPPT) demonstrates efficacy through a dual mechanism of action: physical adsorption of inflammatory mediators (TNF-α, IL-6) and bacterial toxins (elastase, gelatinase), and modulation of the wound microenvironment. Studies show that particles with pore diameters between 40 and 130 µm reduce local inflammatory load by up to 70% [129].

Next-generation smart wound dressings, equipped with electrochemical and optical nanosensors, represent a transformative advancement in complex wound management [130]. These IoT-based medical devices precisely monitor submillimolar levels of critical biochemical parameters [131,132]: tissue pH fluctuations, specific metalloproteinase activity, and microbial load through detection of bacterial signaling molecules (N-acyl homoserine lactones and autoinducer-2) [133]. Integrated with artificial intelligence systems, these dressings not only enable real-time theranostics but also predict critical events, autonomously releasing therapeutics upon detection of established pathological thresholds [134].

Hybrid materials based on clay matrices functionalized with antimicrobial compounds and zwitterionic groups have been extensively investigated due to their multifunctional properties. These systems combine the laminar structure of clay, which provides mechanical and thermal stability, with the synergistic action of biocidal agents and amphoteric molecules, resulting in a material with regenerative capacity, broad-spectrum antimicrobial activity, and modulation of the inflammatory response. The presence of zwitterions enhances biocompatibility and biofilm resistance, while the incorporation of antimicrobial agents potentiates efficacy against pathogens. Furthermore, the geological nature of clay and the scalability of the synthesis process confer significant economic advantages to these materials while maintaining high performance in biomedical applications and tissue engineering [135]. Table 1 shows the comparison between the different types of antibiofilm therapies.

**Table 1:** Comparison of the mechanisms of antibiofilm action between advanced, emerging and innovative therapies.

Therapy	Classification	Mechanism of Action	References
Silver nanoparticles (AgNPs)	Advanced	Release of silver ions, oxidative damage to biofilm	[114]
Liposomal antibiotics	Advanced	Controlled release and biofilm targeting	[119]
Photodynamic Therapy (aPDT)	Advanced	Generation of reactive oxygen species via light and photosensitizer	[122]
Dornase alfa (DNase)	Innovative	Degradation of extracellular DNA from the biofilm matrix	[110]
Nitric oxide (NO) nanoparticles	Emerging	Biofilm dispersion and inflammatory modulation via NO	[123]
Bacteriophages	Emerging	Bacterial lysis and synergism with antibiotics	[126]
Photomodulated NO hydrogels	Emerging	Light-induced NO release	[124]
Microporous particles (MPPT)	Emerging	Passive removal of bacterial toxins and enzymes	[129]
Enzybiotic (phage endolysins)	Emerging	Specific enzymatic degradation of the bacterial wall	[128]
Dressings with smart sensors	Emerging	Biomarker monitoring and adaptive release	[130]
Silica nanoparticles with lectins	Emerging	Targeted delivery of antimicrobials to the biofilm matrix	[136]
Clay dressings with zwitterions	Emerging	Sustained release of antimicrobials with clay support	[135]

## 5. Innovative Therapies and Technological Advances in Wound Healing

Regenerative medicine is an emerging approach that aims to promote the healing of complex wounds using native or synthetic human cells, tissues, and organs [137]. Its application is especially relevant in cases such as diabetic foot ulcers, where therapeutic failure can lead to amputation. Unlike other organisms with a high regenerative capacity, humans only exhibit this ability during embryonic development [138]. However, advances in the field have identified key factors that can be exploited therapeutically. Tissue engineering complements this approach through the development of biomaterials; however, these may present adverse effects. Alternatively, stem cell-based therapies, including the use of embryonic and induced pluripotent stem cells show great promise in overcoming the limitations of conventional methods and enhancing tissue regeneration. Recent advances, such as skin organoids, artificial intelligence–assisted analysis, and 3D bioprinting technologies, further expand the potential of regenerative strategies by enabling precise modeling, prediction, and fabrication of complex tissue architecture [138].

### 5.1. Biomaterials and Scaffolds for Tissue Regeneration

Wound healing of skin lesions is a complex process that involves the interaction between the extracellular matrix and several cell populations such as fibroblasts, keratinocytes, endothelial cells, macrophages and platelets [139]. The process also involves a coordinated effort of several growth factors, cytokines and chemokines, and chronic wounds, those that do not heal, can be formed when there are derangements in the cellular and molecular signals involved in the wound repair mechanisms [14]. In the treatment of skin wounds, surgeons use either full-thickness or split-thickness autologous skin grafts. Split-thickness autologous grafts use a thin layer of skin, including the entire epidermis and part of the dermis, obtained from a donor site [140]. The donor site represents another region of the body such as the ventral thigh, buttocks or upper arm. This therapeutic approach is limited by the availability of the donor site, which has motivated researchers to search for tissue-engineered skin substitutes to improve the results of cutaneous healing [141].

The main objective of skin tissue engineering is to obtain high-quality skin replacement products to facilitate and speed up wound repair. Among the skin replacement therapies, allografts stand out, in which tissues or organs are transplanted from a donor of the same species as the recipient, and autografts, which is a graft of tissue derived from and grafted onto the same individual [142]. Therapeutic advances include the use of biomaterials as scaffolds or temporary biological structures to aid in wound repair. Scaffolds can come in a variety of forms, such as porous structures, fibrous structures, microspheres, hydrogels, composites, and acellular structures. These biomaterials provide a microenvironment necessary for cell growth and differentiation that is analogous to the natural microenvironment of cells, aiding in the healing process [142].

Among the main properties of biomaterials used in tissue engineering, biocompatibility, biodegradability, porosity, mechanical strength and scaffold structures stand out. Biocompatibility refers to the ability of the biomaterial to interact with biological systems without causing damage, adverse reactions or any negative impact on biological entities [143,144] Biodegradability ensures that the biomaterial will decompose slowly over time, concomitantly with tissue regeneration, without generating components that are toxic to cells [145]. Porous biomaterials facilitate cell infiltration, allow nutrient diffusion and oxygenation, and remove waste. The mechanical resistance of biomaterials is related to their ability to withstand mechanical stress without altering their shape. Furthermore, the flexibility of the material allows it to adapt to the shape of the wound. Scaffold structures are essential for determining cell growth and tissue regeneration, due to important factors such as architecture, interconnectivity, hydrophobicity, and hydrophilicity [146].

Scaffold structures can be obtained from materials of synthetic or natural origin or in the form of composites. Synthetic biomaterials, which may not be biodegradable, are among the most widely used in tissue engineering. Among them, a group of aliphatic polyesters stand out-flexible, easy-to-process, non-toxic materials with excellent mechanical strength. These polyesters are a category of polymers formed by repeating units derived from synthetic non-aromatic hydrocarbons. Common examples include polylactic acid (PLA) and polyglycolic acid (PGA) [147]. These biomaterials have been successfully applied in wound healing and as vehicles for the controlled release of growth factors, promoting skin regeneration. Wang and collaborators studied PLA scaffolds combined with nano-hydroxyapatite (nHA) using fused deposition modeling (FDM) technology to create customized porous scaffolds for bone repair [148]. The scaffolds demonstrated good bioactivity, adequate compressive strength, and promoted the formation of bone-like apatite in vitro degradation experiments. The biocompatibility and osteogenic induction properties were proven to be superior to those of the pure PLA scaffold [148].

Natural biomaterials such as collagen, silk, gelatin, and fibrinogen have been widely used in tissue engineering due to their similarity to the native extracellular matrix (ECM), which contributes to improved skin healing. Collagen is one of the most widely used natural biomaterials, possibly due to its abundance in skin and other body tissues [149]. In addition, natural biomaterials of animal origin, such as keratin, bovine serum albumin and eggshell membrane, have also been explored for their beneficial properties in tissue regeneration [150]. Biomaterials derived from animal sources, such as keratin, have demonstrated significant benefits in wound healing, in part due to their ability to release growth factors that promote tissue regeneration. They have antimicrobial and anti-inflammatory properties, in addition to being biocompatible and biodegradable. Examples of composite biomaterials with potential to promote cell adhesion and proliferation involve the combination of cellulose and chitosan nanoparticles with poly(methylmethacrylate) (PMMA) fibers. These structures can act as effective anti-infective dressings against bacteria such as *Staphylococcus aureus* [151].

Martin and collaborators studied a biocompatible scaffold to support the development of neural lineage cells, specialized neurons/glial cells belonging to the CNS/PNS, which replace damaged or lost tissues. The fabricated and freeze-dried scaffolds consisted of biocompatible, natural and synthetic polymers: gelatin and polyvinylpyrrolidone. The 3D scaffold maintained good swelling proficiency, maintaining the intact structure for cell proliferation. Study demonstrated that the gelatin-rich construct favors the prolonged proliferation of stem cells and multiple neurons, along with their plasticity [152].

Motivated by the enormous potential of hydrogels in regenerative medicine, Pasini and collaborators developed novel biocompatible gelatin-based hydrogels that were developed through a green process using polyethylene glycol diglycidyl ether as a crosslinking agent, adding carrageenan and chitosan polysaccharides to the network to better mimic the hybrid composition of the native extracellular matrix [153]. The hydrogels generally present adequate structural stability, high porosity and pore interconnectivity, good swelling and biocompatibility. Human umbilical cord-derived mesenchymal stem cells (hUC-MSCs) were used to test the hydrogels as potential carriers for cell delivery in tissue engineering. The hUC-MSCs cultured within the hydrogels present homogeneous distribution and maintain their growth and viability for at least 21 days of culture, with an increasing tendency of proliferation [153].

To improve the mechanical properties of scaffolds produced from biopolymers, Shan and collaborators incorporated cellulose nanocrystals (CNC) into a calcium-crosslinked sodium alginate/gelatin (SA/Ge/CNC) scaffold to enhance its physicochemical properties. In vitro cytotoxicity and cell growth assays using mouse embryonic fibroblast cells validated the SA/Ge/CNC scaffold as nontoxic and stimulating cell adhesion and proliferation. Furthermore, in vivo skin regeneration experiments using the SA/Ge/CNC scaffold group demonstrated an improved skin wound healing process with accelerated re-epithelialization, increased collagen deposition, and faster extracellular matrix remodeling [154].

### 5.2. Gene Therapy and Cell-Based Treatments for Wound Regeneration

Gene therapy involves introducing genetic material into recipient cells to correct or replace compromised cellular functions. This approach was originally considered a therapeutic option for patients with congenital defects in metabolic functions or in advanced stages of malignant diseases [155]. In recent years, skin tissue regeneration has become a significant focus of gene therapy research. This is due in part to the ease of harvesting and culturing fibroblasts and keratinocytes, allowing in vitro gene transfer assays and the use of these cells as carriers for gene transfer. In addition, skin is readily accessible, and the effects of therapy can be monitored repeatedly, facilitating the assessment of the efficacy and safety of treatments [156].

Approaches aimed at introducing and expressing exogenous DNA into host cells include gene therapy, in which permanent DNA insertion occurs, and gene medicine, which is used for transient transformation and short-term expression of a gene product [157]. Introduction of genes, which requires the selection of an appropriate vector, can occur in vivo, where there is direct introduction of genes into the target tissue, or ex vivo, which is based on the isolation and cultivation of selected cells, they’re in vitro transfection and subsequent transplantation into a host [158].

Vectors of viral and non-viral origin are commonly used in gene transfer. Gene transfer by viral vectors relies on the ability of viruses to introduce and express their own genes within target cells [159]. The process of generating these vectors for gene therapy begins with the modification of the viral genetic material. In this step, the genes responsible for the replication or assembly of the original virus are removed and replaced by the therapeutic gene of interest. To enable the production of modified viruses, special cells called “packaging cells” are used, which are designed to provide the viral functions lost due to the deletion of the original genes [160]. Vectors used in gene therapy are created by modifying different types of viruses. Retroviruses and lentiviruses are examples of viruses that replicate without causing immediate destruction of the host cell, as they form from the membrane of that cell, preserving its structure. The process of lytic replication involves the destruction of the infected cell, releasing new virions into the environment [156].

One of the advantages of non-viral gene therapy is that it does not require the use of viral vectors, which reduces both the risk of infection, and the costs associated with the production of these vectors. Furthermore, the fact that gene expression is temporary can be advantageous in treatments aimed at tissue regeneration, such as wound healing. Among non-viral gene therapies, approaches such as naked DNA delivery using physical methods, such as electroporation and gene gun, and delivery mediated by a chemical carrier, such as cationic polymer and lipid, stand out [161].

Chesnoy and Huang optimized several parameters to obtain reproducible and high-level gene transfer into mouse skin. Older mice showed a significant decrease in gene expression compared to younger mice, and gene expression in the skin was strongly affected by the composition of the solvent used as a carrier. Higher gene expression was obtained when the naked DNA was dissolved in isotonic phosphate-buffered saline compared to isotonic dextrose solution. Finally, transfection efficiency in older mice was significantly improved by increasing the ionic strength of the solvent carrier, being most evident in subdermal smooth muscle cells and epidermal cells [162].

Polycationic polymers can be efficiently used for cellular uptake and endosomal escape, since they form compact, smaller complexes with plasmid DNA and carry amine groups, which confer a positive charge and buffering capacity that allow safe escape from the endosome/lysosome [163]. Among the limitations of the use of polycationic polymers, the possibility of aggregation and eventual toxicity stands out. Thus, smart polymers called “stimulus-responsive polymers” appear to be promising non-viral vectors intended for gene expression specific to location, time and duration [163,164,165]

Human skin cells, such as fibroblasts and keratinocytes, have been used alone or in combination with nanostructured biomaterials to promote wound healing. Fibroblasts produce components of the dermal extracellular matrix, while keratinocytes act as an epidermal barrier [12]. The application of these cells provides bioactive signals essential for skin regeneration. Bioengineered products, such as Epicel, Dermagraft^®^, and Apligraf^®^, combine these elements for epidermal, dermal, or bilayer regeneration, using biodegradable and biocompatible matrices that promote tissue integration and functionality [166].

Several studies have investigated the use of stem cells from embryonic, fetal, and adult tissues for wound healing. Epidermal stem cells and their progenitors, located in terminal hair follicles and the basal layer of the epidermis, have potential for autologous therapies in chronic wounds. However, the limited availability of these cells has led to the use of allogeneic stem cells, especially mesenchymal stem cells (MSCs) [167]. These multipotent cells, derived from various adult tissues such as bone marrow and adipose tissue, can differentiate into multiple cell types, including skin cells, and possess immunomodulatory properties due to their low expression of major histocompatibility complex class II (MHC-II) molecules [168].

Among the sources of stem cells intended for modulating the healing response of acute and chronic wounds, mesenchymal stem cells (MSCs) derived from bone marrow (BM-MSCs), adipose tissue (AD-MSCs) and umbilical cord (UC-MSCs) stand out, as well as induced pluripotent stem cells (iPSCs), which have been investigated for their therapeutic potential in healing [169]. Furthermore, advances in cellular reprogramming have enabled the generation of human induced pluripotent stem cells (hiPSCs) from genetically modified skin fibroblasts containing pluripotency-associated transcription factors (Myc, Oct4, Klf4, and Sox2). hiPSCs represent a potentially inexhaustible source for obtaining functional fibroblasts and other cell lines for clinical application. However, further studies are still needed to ensure the safety and efficacy of these cells before their widespread therapeutic adoption [169]

Platelet-rich plasma (PRP) is widely utilized as a bioactive scaffold in tissue engineering and cell-based therapies. Derived from the patient’s blood via centrifugation, PRP concentrates platelets that release growth factors, cytokines, and proteins essential for cell proliferation, angiogenesis, and collagen synthesis. These bioactive molecules are mainly stored in α-granules, while dense granules and lysosomes aid in platelet activation, antimicrobial defense, and tissue remodeling [170].

The composition of PRP varies according to the preparation method, resulting in different classifications, such as LP-PRP, LR-PRP, P-PRP, and PRF. Platelet-rich fibrin (PRF) stands out for its sustained release of bioactive factors, making it especially promising in surgical and non-surgical procedures. Cervelli and collaborators evaluated the effects of the use of Enhanced Stromal Vascular Fraction (e-SVF) and Fat Grafting with Platelet Rich Plasma (PRP) in regenerative surgery for post-traumatic lower limb ulcers in 20 patients aged between 23 and 62 years. The authors showed that wounds treated with e-SVF healed better (98% re-epithelialization) than those treated with hyaluronic acid (88% re-epithelialization) after 9.7 weeks of treatment. The research also showed that patients treated with PRP and fat grafting also had improved re-epithelialization (98%) when compared to the control group (PRP only) (89%) after 9.7 weeks of treatment [171].

### 5.3. Three-Dimensional Printing and AI in Wound Management

The biomaterials industry has shown substantial growth in recent years, driven mainly by the need to develop materials that mimic human skin and can thus assist in the treatment of complex wounds [172]. The use of biomaterials as skin dressings can partially compensate for the limitation caused by the use of skin grafts (shortage of autologous skin and immunological rejection of allogeneic skin), since they can significantly improve patient care after debridement, providing a moist environment for the wound, ensuring sterility and gas exchange, promoting angiogenesis and regeneration of connective tissue and can also be easily removed after healing [173].

In the last decade, 3D bioprinting has emerged as a technology for the elaboration of complex structures obtained by layer-by-layer deposition of cell-laden biotins [174]. Three-Dimensional printing involves a variety of techniques such as stereolithography, microextrusion, laser-assisted printing or inkjet printing. In this sense, the appropriate composition of bioinks using a variety of materials allows the bioprinting of constructs with various architectures, mechanically stable [172].

Biotins are composed of biocompatible and biodegradable materials, which can be of natural or synthetic origin. Natural components include hyaluronic acid, alginate, collagen, chitosan, chitin, cellulose, thrombin, etc. Synthetic materials include ε-caprolactone, polylactic acid, polyglycolic acid, polyurethanes, polycarbonates, trimethylene carbonate, polyethylene glycol methacrylate, and polypropylene fumarate [172,175]. When intended for the healing process, these materials include active substances such as antibiotics or peptides, often combined with growth factors, to facilitate the stimulation, growth, proliferation and migration of cells during the healing process [176].

Intini and collaborators evaluated the biocompatibility, cytocompatibility and toxicity of 3D printed chitosan porous scaffolds for human fibroblasts (Nhdf) and keratinocytes (HaCaT). The 3D cell cultures obtained after 20 and 30 days of incubation exhibited significant qualitative and quantitative cell growth in vitro, with the best cell growth obtained after 35 days being achieved in the 3D scaffolds in which Nhdf and HaCaT cells, seeded together, filled the pores in the scaffolds. The formation of an initial skin-like layer consisting of fibroblasts and keratinocytes growing together was also observed. The application trials of 3D printed scaffolds in wound healing performed in diabetic rats demonstrated that they improve the quality of the restored tissue when compared to both commercial dressings and spontaneous healing [177]

Xu and collaborators studied the physicochemical and biological properties of scaffolds based on 2,2,6,6-tetramethylpiperidine-1-oxyl radical, cellulose nanofibrils (CBFs) and gelatin methacrylate (GelMA). The assays involving cell culture with 3T3 fibroblasts showed non-cytotoxic and biocompatible characteristics for both the formulated inks and the printed scaffolds. The study also showed that GelMA incorporated into the CNF hydrogel promoted the proliferation of fibroblasts, evidencing the potential of the scaffolds obtained in 3D printing in applications involving wound healing [178].

Artificial intelligence (AI) is an emerging technology that aims to simulate and enhance human intelligence, with a growing impact on healthcare. Various tools, such as machine learning (ML), neural networks (NN), deep learning (DL), and electronic health records (EHR), enable the processing of large volumes of complex medical data, improving analysis, interpretation, and clinical decision-making. In wound diagnosis, AI integrates medical images (such as MRIs, CT scans, and 3D ultrasounds) to automate and improve the diagnostic process, as well as optimize healthcare resources [176].

Recent advances in artificial intelligence (AI) have made it possible to substantially improve the accuracy of wound assessment [179]. AI-based technologies offer greater accuracy not only in measuring wound dimensions but also in analyzing wound topography, identifying edges, and quantifying the ratio between different tissue types, thus increasing the rigor and reliability of clinical analyses aimed at wound closure [176].

The effectiveness of artificial intelligence applications in wound assessment has been demonstrated by several studies. Chino and collaborators developed a semi-automatic approach, called Automatic Skin Ulcer Region Assessment (ASURA), a deep neural network-based system capable of segmenting wounds with over 90% accuracy using the Dice score and estimating pixel density within 5%. Using a semi-automatic approach, it also calculated wound area with a 14% error, outperforming current methods by up to 16% [180].

In another proposal, Zhao and collaborators used a vision-laser scanner developed for 3D reconstruction of wound edges and topology, combining measurements from a laser sensor with encoder data and a 2D camera. The system uses an artificial neural network to estimate additional points, speeding up the scanning process. Tests showed high accuracy in locating the wound edge, with mean errors below 0.61 mm. The scanner simultaneously generates point clouds of both the wound skin and its edges, improving precision in wound closure for clinical applications [181].

Intelligent robotic systems with minimal or no external intervention are becoming a reality, especially in medicine. The shortage of skilled professionals and the increase in chronic wounds are driving the adoption of these technologies. Fully automated medical stations for wound analysis will allow the objective collection of measurements such as circumference, area, and volume. The initial development of this advancement involves creating robust robotic systems capable of performing accurate 3D reconstruction of wound surfaces [182].

The Internet of Things (IoT) enables real-time monitoring using IoT-enabled sensors and smart dressings capable of monitoring wound temperature and humidity levels, aiding in the effectiveness of therapies [183]. Ramachandram and co-workers conducted an inter- and intra-rater agreement study using 58 anonymized images of chronic wounds from the Swift Medical Wound Database. The images were divided into three subsets with 50% overlap to assess intra-rater consistency. Five experts labeled four tissue types (epithelial, granulation, slough, and eschar) weekly using an online tool. Two deep convolutional neural networks were developed to segment wounds and tissues, trained on 465,187 and 17,000 image-label pairs, respectively. The models demonstrated high accuracy, good performance across different imaging conditions, were free from skin tone bias, and operated in near real-time on mobile devices. The study showed that inter-rater agreement in identifying chronic wound tissues ranged from low to moderate [184].

Singh and collaborators evaluated the association of single nucleotide polymorphisms with increased risk of diabetic foot ulcer (DFU) in patients with type 2 diabetes mellitus (T2DM). The researchers used multivariate linear regression (MLR) and artificial neural network (ANN) modeling to observe their predictability for the risk of DFU in patients with T2DM and concluded that the ANN model (83%) was superior to the MLR model (76%) and can be used as a tool for assessing the risk of DFU in patients with T2DM [185].

Cho and collaborators developed a model to predict chronic wound healing based on the analysis of electronic medical records for 620,356 chronic wounds of various origins, belonging to 261,398 patients treated at 532 specialized wound care clinics in the United States [186]. The factors related to both the patient and the wound that impact the healing process were selected based on previous studies and the expertise of medical professionals. From this, predictive models were created using logistic regression and decision trees, with the objective of estimating the chance of wound healing within a period of 12 weeks. These models were trained with 70% of the randomly selected wounds and subsequently evaluated using the remaining 30% of the data. The results indicated that 365,659 wounds (equivalent to 58.9% of the total) presented healing in this time interval. The models performed reasonably well, with an area under the curve (AUC) of 0.712 for the logistic model and 0.717 for the decision tree-based model. Specific wound characteristics such as location, size, depth, and type were more relevant predictors than patient demographics or comorbidities [186].

### 5.4. Cutaneous Organoids in Wound Regeneration

Organoids are three-dimensional structures derived from stem cells that highly replicate the organization and physiological functions of human tissues in vitro. Compared to two-dimensional cultures, organoids exhibit multiple cell types and complex interactions, making them superior models for biomedical research. With advances in biofabrication and tissue engineering, organoids of various organs, including skin, have been successfully developed [187].

Cutaneous organoids offer significant advantages over traditional skin models by self-organizing and differentiating into complex structures, such as hair follicles and sebaceous glands. These models more closely resemble real human skin and are ideal for studying wound healing mechanisms. Furthermore, they hold great potential in regenerative medicine, enabling the development of more effective therapies for skin lesions [188].

Wounds resulting from trauma, burns, or surgery present significant challenges for skin regeneration, especially in cases of difficult healing or loss of adnexal structures such as hair follicles and sweat glands. In this scenario, skin organoids emerge as a promising approach in regenerative medicine, due to their ability to mimic the three-dimensional structure and functionality of human skin [188].

Most skin organoids used in research are derived from induced pluripotent stem cells (iPSCs). Studies have shown that these organoids can differentiate into multiple cell types and form complex structures, such as stratified epidermis, dermis with adipose tissue, and functional skin appendages [189]. When transplanted into animal models, these organoids demonstrated the ability to integrate with host tissues, inducing hair follicle formation, vascular and glandular regeneration, and promoting efficient epithelialization [190].

Other strategies include the use of cell co-cultures encapsulated in hydrogels (such as endothelial and mesenchymal cell spheres), as well as organoids derived from epithelial cells or reprogrammed keratinocytes. These approaches have demonstrated efficacy both in vitro and in vivo in burn and full-thickness wound models [191].

In difficult-to-heal wounds, such as diabetic ulcers, organoids generated by transdifferentiation of mesenchymal stem cells overexpressing chemokine receptors also promoted dermal epithelialization and vascularization, accelerating wound closure. These results indicate that cutaneous organoids, combined with cellular bioengineering, represent a powerful and versatile tool with great translational potential for the treatment of complex skin lesions [192].

Advanced biotechnologies, such as organoids, multiphysiological systems, and organs-on-a-chip, have demonstrated performance equal to or superior to traditional animal models, offering promising alternatives for biomedical applications [193]. In the context of skin regeneration, FOXC1 overexpression has been shown to be effective in reprogramming epidermal cells into sweat gland-like cells, promoting their regeneration and wound healing [194]

Skin organoids have also been used in modeling skin tumors—such as basal cell carcinoma, squamous cell carcinoma, melanoma, and Merkel cell carcinoma—as well as in drug screening and the study of therapeutic resistance [195]. Furthermore, patient-derived or genetically edited hiPSCs have enabled the simulation of genetic skin diseases, such as epidermolysis bullosa, with promising results obtained through gene editing via CRISPR/Cas9 [196].

## 6. Benefits of Using Peptides in Nanoparticles

Conjugated to nanoparticles, peptides tend to maintain their biological activity while being protected from enzymatic degradation, enabling sustained and targeted release in injured tissues (Table 2). Among nanostructured systems, chitosan nanoparticles (CSNPs) functionalized with peptides have stood out in accelerating wound healing.

Recent studies have shown that CSNPs encapsulated with growth factors, such as EGF, protect the protein from degradation, promote controlled release and increase the rate of wound closure in animal models [197]. In addition, the modification of CSNPs with antimicrobial peptides, such as P5S9K, associated with recombinant growth factors, has shown synergistic potential in promoting tissue regeneration and reducing wound infections [198].

**Table 2:** Overview of peptide-loaded/adsorbed nanostructures for antimicrobial and wound healing applications.

Type	Peptide/AMPc	Diameter (nm)	EE%	Concentration	Application	Outcome	References
Gold nanoparticles (AuNPs)	LL37	10 ± 1	ND	100 μg/mL	Therapy for diabetic wound healing	AuNPs@LL37 showed superior antibacterial action, synergistic effect with promotion of angiogenesis and accelerated healing of diabetic wounds, in addition to high biocompatibility in vitro and in vivo.	[199]
PLGA	Plectasin	224 ± 3 to 215 ± 3	71–90	500 μL of peptide stock solution to PLGA solution (60 mg/mL)	Antimicrobial activity against *Staphylococcus aureus*	Plectasin-loaded nanoparticles demonstrated greater efficacy than free plectasin, without affecting the viability of eukaryotic cells at the concentrations tested.	[200]
Chitosan	Octominin	372 ± 2	96.4	Octoprohibitin (1 mg/mL)	Bacterial and fungal infections	Nanoencapsulated octominin demonstrated greater antimicrobial activity against *C. albicans* and *A. baumannii* compared to free Octominin.	[201]
PLGA	GIBIM-P5S9K (G17) and GAM019 (G19)	1022 ± 3 and 1976 ± 4	41–67	10% PLGA (*w*/*v*) in ethyl acetate (EtAc) in a ratio of peptide solution to PLGA dispersion of 2:1	Antimicrobial activity against *Staphylococcus aureus* (MRSA) and *Escherichia coli*	Nanoparticles loaded with G17 and G19 peptides showed slow release and bacteriostatic potential against *E. coli* O157:H7 and MRSA.	[197]
Chitosan	CAMA-CPP	597 ± 1	75.2	CS solution (0.50% *w*/*v*); Acetic acid (1%) and STPP (0.25%) solution. CAMA-CPP was added at a concentration of 0.10 mg/mL to the CS solution.	Antimicrobial activity against *Salmonella enteritidis*	ENC CAMA-CPP demonstrated pH-dependent sustained release, enzymatic and biological stability, in vitro safety, and antimicrobial and immunomodulatory efficacy against multidrug-resistant *S. enteritidis*.	[202]
Chitosan/alginate	Pexiganan (MSI-78)	ND	ND	*PNPs* 32 µg/mL	Helicobacter pylori infections	PNPs improved peptide stability in the stomach and demonstrated more effectiveness in eradicating *H. pylori* in the stomach of rats compared to pexiganan	[203]
Chitosan	Ultra short AMP (RBRBR)	121 ± 1	51.3	Chitosan 1.75% (*v*:*v*); acetic acid (1 mg/mL, 5 pH); TPP (1 mg/mL) and 500 µg of RBRBR.	Antimicrobial activity	RBRBR-CS-NPs demonstrated prolonged and selective action against several Gram-positive bacteria, including resistant strains of *S. aureus*	[204]
Solid Lipid Nanoparticle (SLN)	LL-37 and SERPINA A1	261 ± 4	83.3	8.48 μg for LL37 and 43.5 μg for A1 per mg of SLNs and 16.32 μg for LL37 and 62.47 μg for A1 per mg of SLNs	Wound healing and antimicrobial activity	LL37-A1-SLNs accelerated the wound healing process and improved antibacterial activity against *S. aureus* and *E. coli* compared with LL37 or A1	[205]
Lipid-coated mesoporous silica nanoparticles	PA-targeting LL-37 peptide	620 ± 10	79.3	MSNs were loaded with Col by mixing 20 mL of MSN (10 mg/mL) with Col (20 mg, 17 μmol)	Antimicrobial activity against *Pseudomonas aeruginosa*	The Col@MSN@LL-(LL-37) nanocomposite demonstrated a 6.7-fold enhancement in antimicrobial activity relative to free Col	[206]
Chitosan	Antimicrobial Peptide Octoprohibitin	246 ± 1	34.2	1 mg of Octominin-containing Octominin-CNPs	Antimicrobial activity against *Acinetobacter baumannii*	Octoprohibitin encapsulated in carbon nanoparticles (CNPs) exhibited potent antimicrobial activity against multidrug-resistant A. baumannii, effectively targeting both planktonic cells and established biofilms	[207]
PLGA	LL37	304.5 ± 10.0	70.2	20 mg of PLGA to 20 μg of LL37 (95.0% pure, Caslo ApS, DK)	Healing and antimicrobial activity against *Escherichia coli*	PLGA-LL37NP nanoparticles enhanced wound healing activity and significantly increased IL-6 and VEGFa at the mRNA level improving angiogenesis	[77]
Nesoporous polydopamine (MPDA)	RL-QN15	205	67.3	1 mg MPDA was dispersed in RL-QN15	Therapies for the treatment of skin wounds	MPDA-RL-QN15 nanocomposites demonstrated up to 50-fold enhanced wound healing activity in animal models, with efficacy confirmed through histological analysis.	[208]
Hollow Silica Nanoparticles Loaded with RL-QN15 Peptide	RL-QN15	50	ND	HPDA was mixed with Cy_RL-QN15_ (1 nM)	Therapeutic strategy for clinical chronic skin wound healing.	The HPDAlCyRL-QN15/ZA hydrogel accelerated diabetic wound healing by promoting cell regeneration, angiogenesis, collagen deposition, and by reducing inflammation and oxidative stress.	[209]

ND: Not described.

In addition, the incorporation of peptides into hydrogels and nanocomposite films has been explored as a form of localized topical application. These materials combine the advantages of nanoparticles with physical support to protect the wound, maintain humidity and facilitate the gradual release of bioactive agents, resulting in an ideal environment for tissue regeneration [210]. Approaches involving nanoparticles that are sensitive to stimuli, such as pH, temperature or inflammatory enzymes, have also enabled ‘on-demand’ release of peptides, increasing therapeutic precision.

In summary, the conjugation of therapeutic peptides to nanoparticles represents a multifunctional and highly effective approach to wound treatment. By integrating bioactive properties, molecular protection, tissue penetration capacity and controlled release, these nanostructured systems have the potential to redefine the paradigms of regenerative medicine, promoting more effective, safe and personalized interventions.

### 6.1. Types of Nanoparticles Used for Peptides

A major challenge in peptide encapsulation is preserving their structural stability and bioactivity, as they are susceptible to hydrolysis by gastrointestinal proteases and peptidases. Evidence suggests that most peptides degrade during digestion, with around 40 enzymes including gastric pepsin and intestinal alkaline proteases contributing to their potential inactivation upon oral administration [211].

In this context, nanostructured systems of different types can be employed to encapsulate these bioactive peptides, with the choice of nanoparticles depending on the application. This selection is critical, as it requires a thorough understanding of multiple factors that influence the loading efficiency, stability, and controlled release of peptides whether they are incorporated within or adsorbed onto the nanostructured system [212].

A variety of nanoparticle classes have been widely explored as carriers for the controlled delivery of bioactive peptides. Polymeric nanoparticles are among the most prominent, including those derived from natural polymers such as chitosan, alginate, and gelatin, as well as synthetic polymers like poly(lactic-co-glycolic acid), poly(ε-caprolactone), poly(lactic acid), and polyvinyl alcohol. Inorganic nanomaterials, particularly mesoporous silica nanoparticles and carbon nanotube-based structures, have also shown considerable promise for peptide encapsulation and targeted release, as summarized in Table 2.

**Figure 4:**
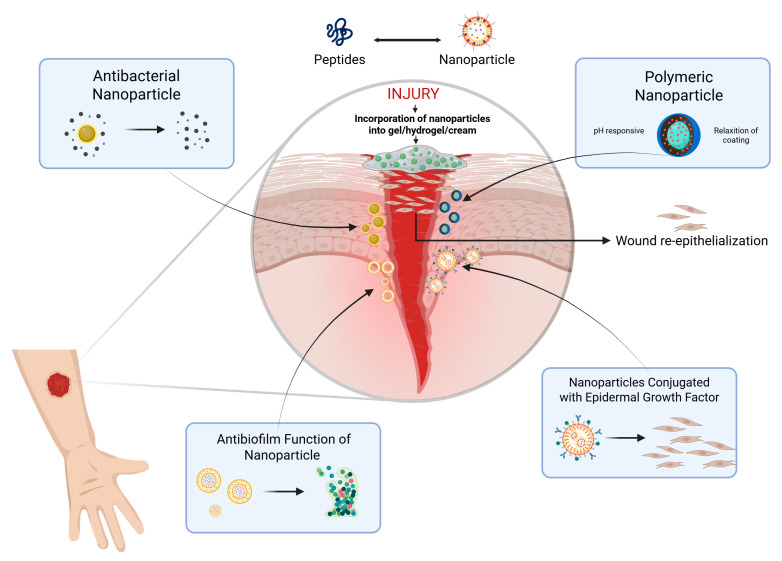
Schematic representation of different types of nanoparticles designed for wound healing and antibacterial applications, incorporating bioactive peptides to enhance antimicrobial activity and tissue regeneration. Created in BioRender, Academic Individual License granted to UCD/E. B. Souto OBRSS Research Support Scheme (AIUCD241022-cd0d). Souto, E. (2025) https://BioRender.com/hn8vnqn (accessed on 1 August 2025).

The application of nanotechnology in peptide delivery has shown highly promising results, particularly in enhancing wound healing processes (Figure 4). These nanomaterials exhibit a synergistic effect, combining low hemolytic activity, minimal toxicity, high specificity, and improved bioavailability key attributes that make them attractive for therapeutic use [213]. The wound healing process encompasses a series of intricate cellular and molecular stages. In chronic wounds, for instance, the excessive release of proteolytic enzymes and ROS disrupts the delicate balance between tissue degradation and maturation. This imbalance alters the redox equilibrium, ultimately impairing cellular repair mechanisms and hindering the overall healing process [214].

The selection of peptides for nanoparticle conjugation is influenced by multiple factors, including the physicochemical properties of the nanomaterial, surface ligands, and peptide sequence. Particular attention must be paid to functional groups, especially when the peptide contains primary amines that are essential for biological activity. In such cases, conjugation strategies should preserve these critical functional groups to maintain the peptide’s therapeutic efficacy [185,214,215].

Multiple approaches are employed for conjugating peptides to nanoparticles, each utilizing specific molecular interactions. Electrostatic interactions represent one fundamental strategy, where complementary charges between the peptide and nanoparticle surface enable efficient binding. Another approach involves direct interactions between peptide functional groups and the nanoparticle surface, as exemplified by thiol-containing peptides that form stable bonds with gold nanoparticles (AuNPs) through covalent Au-S linkages. For enhanced specificity, secondary interactions such as the high-affinity biotin-streptavidin system allow for precise and oriented assembly of peptide-nanoparticle complexes. Finally, covalent conjugation represents another key mechanism, achieved through established bioconjugation reactions. These include: (1) carbodiimide-mediated coupling (e.g., EDC-assisted reactions between amine and carboxyl groups), and (2) NHS-maleimide chemistry targeting specific functional groups (amines and thiols). These well-characterized reactions enable precise and stable peptide-nanoparticle [216].

Nanostructured systems provide key advantages including biocompatibility, biodegradability, and tunable drug release kinetics, which collectively enhance both antimicrobial and wound healing activities. Moreover, liposomes represent another promising platform due to their amphiphilic structure that enables simultaneous encapsulation of both hydrophilic and hydrophobic compounds [217]. Metallic nanoparticles, particularly gold (Au)- and iron oxide-based systems, have also demonstrated significant potential for peptide delivery. This stems from the unique ability of certain peptides to traverse cellular membranes, which facilitates nanoparticle internalization and enables efficient intracellular delivery of therapeutic agents [218].

Wang and collaborators [219] developed an innovative therapeutic platform combining ultra-functionalized gold nanoparticles with the antimicrobial peptide LL-37 (AuNPs@LL-37) for topical treatment of diabetic wounds. This system synergistically exploited the cationic properties of AuNPs for DNA condensation and the inherent antimicrobial activity of LL-37, achieving both efficient gene delivery and potent bactericidal effects. When loaded with pro-angiogenic VEGF plasmids, the AuNPs@LL-37 complex demonstrated enhanced angiogenesis, effective bacterial infection control, and accelerated wound healing in both in vitro and in vivo models. These multifunctional capabilities highlight its potential as a biocompatible and effective therapeutic approach for chronic wound management, particularly in the challenging context of diabetic wounds with or without bacterial infection.

Solid lipid nanoparticles (SLNs) encapsulating the antimicrobial peptides LL-37 and Serpin A1 were successfully developed by Fumakia and Ho [205]. This work demonstrated that co-encapsulation of both peptides in SLNs produced nanostructures averaging 261 nm in size with high encapsulation efficiency (83%), while achieving sustained and controlled release kinetics. Through in vitro and in vivo testing, the authors observed accelerated wound healing via enhanced fibroblast and keratinocyte activity. Furthermore, the LL-37/Serpin A1-loaded SLNs exhibited potent antimicrobial effects against *S. aureus* and *E. coli*, outperforming either peptide administered alone. These findings underscore the significant potential of peptide-loaded nanocarriers for treating bacterially infected chronic wounds through combined regenerative and antimicrobial mechanisms.

The study by Qin and collaborators developed mesoporous polydopamine nanoparticles (MPDA) functionalized with the RL-QN15 peptide [208]. The results showed conjugation of the RL-QN15 peptide to mesoporous polydopamine nanoparticles (MPDA) yielded nanostructures with an average size of 205 nm and an encapsulation efficiency of 67.3%. The system demonstrated sustained release kinetics, with RL-QN15 being continuously released over 0–16 h, reaching a cumulative release rate of 79.12% at 24 h. In vivo studies revealed significantly accelerated wound healing, showing up to 50-fold enhanced regenerative activity compared to free peptide, as confirmed by histological analyses demonstrating increased fibroblast proliferation and organized collagen deposition. Furthermore, the MPDA-RL-QN15 nanocomposites exhibited synergistic antimicrobial activity against clinically relevant wound pathogens *P. aeruginosa* and *S. aureus*, outperforming standalone RL-QN15. These findings highlight the therapeutic potential of this nanoplatform for treating infected cutaneous wounds by combining regenerative and antimicrobial properties in a single system.

Continuing this line of research, Qin and collaborators designed an innovative multifunctional nanoplatform for enhanced diabetic wound healing by integrating regenerative and antimicrobial properties [208]. The system consisted of mesoporous polydopamine nanoparticles (MPDA NPs), synthesized via a soft-template-assisted method using Pluronic F-127 and 1,3,5-trimethylbenzene in a basic ethanol–water mixture, followed by oxidative self-polymerization of dopamine hydrochloride. The peptide RL-QN15 was then conjugated to the surface of MPDA via Schiff base and Michael addition reactions, resulting in MPDA-RL-QN15 nanocomposites with an average size of 50 nm approximately, with a spherical morphology and hollow structure. The release efficiency of CyRL-QN15 progressively decreased across the formulations CyRL-QN15/ZA, HPDAlCyRL-QN15, and HPDAlCyRL-QN15/ZA. Despite all systems exhibiting release rates greater than 50% within the first 24 h, the cumulative release after 48 h reached 93.51% for CyRL-QN15/ZA, 79.89% for HPDAlCyRL-QN15, and 73.48% for HPDAlCyRL-QN15/ZA.

**Figure 5:**
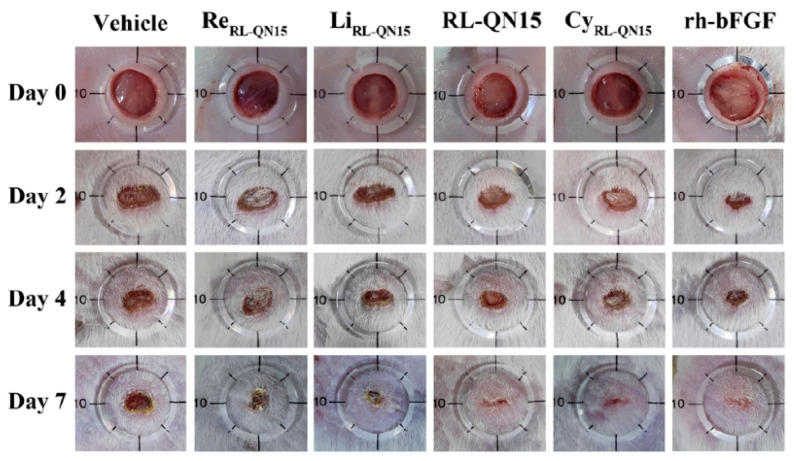
Representative and quantitative plots of the effects of RL-QN15, ReRL-QN15, LiRL-QN15, and CyRL-QN15 on wound healing in Kunming mice with full-thickness skin wounds in vivo [220].

Fu and collaborators [220] continued this line of research by developing an innovative multifunctional nanoplatform for enhanced diabetic wound healing, integrating both regenerative and antimicrobial properties (Figure 5). Histological analyses confirmed up to a 50-fold increase in regenerative activity compared to free RL-QN15 peptide. Furthermore, the nanocomposites showed potent antimicrobial activity against *P. aeruginosa* and *S. aureus*, surpassing the efficacy of the peptide alone. These results demonstrate that the MPDA-RL-QN15/ZA hydrogel represents a promising therapeutic strategy for the treatment of chronic and infected skin wounds by combining sustained drug release, bioactivity, and antimicrobial function in a single nanostructured system.

These findings demonstrate that recent advances in bioactive peptide-nanoparticle conjugation/encapsulation represent an innovative therapeutic strategy for managing chronic and infected wounds. This approach synergistically combines the physicochemical properties of nanoparticles with the biological selectivity and activity of peptides, enabling not only controlled and targeted delivery but also simultaneous enhancement of antimicrobial and pro-regenerative effects. Current evidence highlights accelerated wound healing, significant bacterial load reduction, and improved tissue response modulation, underscoring the technology’s translational potential. However, full clinical implementation requires further rigorous preclinical studies and clinical trials to validate large-scale efficacy and safety.

### 6.2. Clinical Applications

A phase I/II clinical study by Grönberg and collaborators [221] evaluated 34 patients with chronic venous ulcers resistant to healing. Participants were randomized to receive topical applications of LL-37 at concentrations of 0.5, 1.6, or 3.2 mg/mL, or a placebo, applied twice weekly for four weeks. The study revealed that the two lower doses (0.5 and 1.6 mg/mL) significantly enhanced healing rates compared to the placebo, with average ulcer area reductions of 68 and 50%, respectively. Furthermore, no significant local or systemic adverse effects were observed, affirming the safety and tolerability of LL-37 for the topical management of chronic wounds.

In line with these findings, a randomized, double-blind, placebo-controlled study assessed the efficacy of LL-37 cream in promoting the healing of mildly infected diabetic foot ulcers. Patients applied either LL-37 cream or placebo twice weekly for four weeks. The treatment significantly enhanced the granulation index of the wounds compared to placebo, indicating accelerated healing. However, no significant reduction was noted in IL-1α and TNF-α levels or aerobic bacterial colonization, suggesting that LL-37 primarily facilitates healing through non-inflammatory pathways [222].

In a complementary approach, a self-aggregating peptide hydrogel was engineered as a three-dimensional scaffold to support spheroids of mesenchymal stem cells (MSCs) derived from the human umbilical cord. When applied to skin wounds in diabetic mice, this system accelerated healing by modulating inflammation, enhancing angiogenesis, and stimulating tissue regeneration. It maintained cell viability and preserved the three-dimensional microenvironment of the spheroids, fostering the sustained release of pivotal bioactive factors, including VEGF, TGF-β, and IL-10, which are crucial for efficient tissue repair. Notably, the treatment also reduced chronic inflammation and metalloproteinase expression, while promoting new vessel formation and wound re-epithelialization [223].

Building on the theme of stem cell-based therapies, PLGA nanoparticles functionalized with specific peptides to enhance cell adhesion were developed to improve mesenchymal stem cell retention within the wound microenvironment. This strategy resulted in a significant increase in healing rates in diabetic murine models. The nanoparticles offered protection against oxidative stress and enabled controlled release of bioactive factors, crucial for maintaining stem cell viability and function during the regenerative process [224].

A system of gold nanoparticles conjugated with bioactive peptides was developed, demonstrating not only antioxidant capacity but also the ability to modulate the local inflammatory response in chronic wounds. This synergistic effect facilitated the migration and proliferation of fibroblasts and endothelial cells, resulting in accelerated tissue regeneration, a significant reduction in bacterial load, and enhanced angiogenesis [225].

Building on the therapeutic potential of peptide-functionalized nanoparticles, silver nanoparticles conjugated with MT6 and CuTP1 peptides were synthesized using a green approach that avoided toxic reducers, with stabilization achieved directly by the peptides. In a murine model of excisional dorsal wounds, treatment with MT6-AgNPs led to 71.97 ± 4.35% wound closure after 7 days, representing a 5.48-fold improvement (*p* < 0.05) compared to the free peptide. Similarly, the CuTP1-AgNP system promoted 62.37 ± 18.33% closure, 2.82 times greater than free CuTP1. Both formulations also demonstrated antibacterial activity against *S. aureus* and *E. coli*, along with in vitro biocompatibility with human dermal fibroblasts [226].

These findings highlight the significant therapeutic promise of peptide-functionalized metallic nanoparticles as next-generation biomaterials for wound management. By combining regenerative and antimicrobial capabilities within a single nanosystem, such platforms address key challenges in chronic wound healing, particularly under diabetic conditions. Although most results remain at the preclinical stage, the consistent evidence of enhanced tissue regeneration, infection control, and biocompatibility provides a solid foundation for future translational studies. Therefore, clinical trials evaluating the safety, pharmacokinetics, and therapeutic efficacy of these peptide-nanoparticle conjugates are essential to confirm their potential and pave the way for their integration into advanced wound care therapies.

## 7. Conclusions

Wound healing is a complex process involving cellular and molecular interactions, with cytokines and growth factors playing key roles. AMPs offer promising therapeutic potential by modulating immune responses and directly targeting pathogens, especially in chronic wounds where conventional treatments often fail. Bacterial biofilms pose a significant challenge by protecting microbes from antibiotics and immune attacks. Innovative approaches like enzymatic dispersal, chelators, and photodynamic therapy are essential to disrupt biofilms and enhance treatment efficacy. Emerging technologies, particularly nanotechnology, are revolutionizing wound care. Engineered biomaterials mimic the extracellular matrix to support tissue regeneration, while nanomedicine allows for precise delivery of therapeutics, improving healing outcomes. Regenerative medicine strategies, including the use of stem cells and bioengineered organoids, are opening new possibilities for promoting tissue repair and restoring skin architecture, especially in severe or non-healing wounds. Advances like 3D bioprinting and gene therapy also hold great promises for personalized and effective treatments. In summary, combining biological insights with nanotechnology, regenerative medicine, and other innovations paves the way for improved, targeted therapies that can transform wound management and enhance patient outcomes.

## Data Availability

Not applicable.
